# Trastuzumab uptake and its relation to efficacy in an animal model of HER2-positive breast cancer brain metastasis

**DOI:** 10.1007/s10549-017-4279-4

**Published:** 2017-05-10

**Authors:** Gail D. Lewis Phillips, Merry C. Nishimura, Jennifer Arca Lacap, Samir Kharbanda, Elaine Mai, Janet Tien, Kimberly Malesky, Simon P. Williams, Jan Marik, Heidi S. Phillips

**Affiliations:** 1 Genentech, Inc., 1 DNA Way, South San Francisco, CA 94080 USA; 2 Calico Labs, 1170 Veterans Blvd, South San Francisco, CA 94080 USA; 3 Novartis Institutes for BioMedical Research, 250 Massachusetts Ave, Cambridge, MA 02139 USA

**Keywords:** Trastuzumab, Blood–brain barrier, Brain metastasis, Breast cancer, T-DM1

## Abstract

**Purpose:**

The extent to which efficacy of the HER2 antibody Trastuzumab in brain metastases is limited by access of antibody to brain lesions remains a question of significant clinical importance. We investigated the uptake and distribution of trastuzumab in brain and mammary fat pad grafts of HER2-positive breast cancer to evaluate the relationship of these parameters to the anti-tumor activity of trastuzumab and trastuzumab emtansine (T-DM1).

**Methods:**

Mouse transgenic breast tumor cells expressing human HER2 (Fo2-1282 or Fo5) were used to establish intracranial and orthotopic tumors. Tumor uptake and tissue distribution of systemically administered ^89^Zr-trastuzumab or muMAb 4D5 (murine parent of trastuzumab) were measured by PET and ELISA. Efficacy of muMAb 4D5, the PI3K/mTOR inhibitor GNE-317, and T-DM1 was also assessed.

**Results:**

^89^Zr-trastuzumab and muMAb 4D5 exhibited robust uptake into Fo2-1282 brain tumors, but not normal brains. Uptake into brain grafts was similar to mammary grafts. Despite this, muMAb 4D5 was less efficacious in brain grafts. Co-administration of muMAb 4D5 and GNE-317, a brain-penetrant PI3K/mTOR inhibitor, provided longer survival in mice with brain lesions than either agent alone. Moreover, T-DM1 increased survival in the Fo5 brain metastasis model.

**Conclusions:**

In models of HER2-positive breast cancer brain metastasis, trastuzumab efficacy does not appear to be limited by access to intracranial tumors. Anti-tumor activity improved with the addition of a brain-penetrant PI3K/mTOR inhibitor, suggesting that combining targeted therapies is a more effective strategy for treating HER2-positive breast cancer brain metastases. Survival was also extended in mice with Fo5 brain lesions treated with T-DM1.

**Electronic supplementary material:**

The online version of this article (doi:10.1007/s10549-017-4279-4) contains supplementary material, which is available to authorized users.

## Introduction

The incidence of brain metastases in patients with metastatic breast cancer (MBC) appears to be increasing over time, due in part to improved control of systemic disease, prolonged survival, and enhanced detection [1]. Brain metastases lead to substantial morbidity—in patients with MBC, including cerebral edema, headaches, seizures, motor impairment, speech difficulty, and mental disturbances [2]. Systemic therapy has limited efficacy in treating brain metastases, possibly due to poor penetration of the blood–brain barrier (BBB), expression of drug efflux pumps in the BBB, enriched abundance of ErbB ligands, or acquired resistance following treatment with multiple prior regimens [3]. In addition to systemic therapy, standard treatments for brain metastases include whole-brain radiation, stereotactic radiosurgery, and, for eligible patients with solitary lesions, surgical resection [4]. Despite these interventions, median overall survival (OS) is poor, ranging from 3–30 months, depending on breast cancer subtype and treatment [5].

Approximately 20% of all breast cancers overexpress human epidermal growth factor receptor 2 (HER2) [6, 7]. In RegistHER, a prospective, observational study of 1012 patients with HER2-positive MBC, central nervous system (CNS) metastases were documented in 37.3% of patients over a median follow-up of 29 months [8]. Other analyses found brain metastases to be present in up to 55% of patients with HER2-positive MBC [5, 9]. Amplification and/or overexpression of HER2 may play a role in the occurrence or progression of brain metastases. In a mouse model of MBC, HER2 overexpression promoted outgrowth of breast tumor-derived brain metastases, with the number of large brain metastases increasing 3-fold in mice inoculated with high HER2-expressing versus low HER2-expressing human breast cancer cells [10]. In a study of more than 600 patients with MBC, HER2-positivity was a significant and independent risk factor for subsequent development of brain metastases [11]. Moreover, in a study comparing HER2 mRNA levels in unlinked archival brain metastases and primary breast tumors, HER2 mRNA was found, on average, to be 5-fold more abundant in brain metastases than in primary tumors [10].

HER2-targeted agents, such as trastuzumab [8, 12], lapatinib [13, 14], and trastuzumab emtansine (T-DM1) [15, 16], have been shown to improve outcomes in patients with HER2-positive MBC and CNS metastases, including leptomeningeal or brain parenchymal lesions [7, 8]. In registHER, patients administered trastuzumab exhibited a median OS of 17.5 months from the date of CNS disease diagnosis compared with 3.8 months for patients not receiving trastuzumab [8]. Moreover, multivariate analysis showed trastuzumab to be a significant independent predictor of survival [8]. In another retrospective study of women with HER2-positive MBC and CNS metastases, median OS was 11.6 months among those who received trastuzumab at the time of, or prior to, CNS lesion diagnosis compared with 6.1 months among women who did not receive trastuzumab (*p* = 0.03) [12]. It is unclear, however, whether the improvements in OS stem from control of systemic, extra-cranial disease, or from direct effects of trastuzumab on brain lesions.

Although efficacy of systemic therapy for treating brain metastases may be limited by the inability of HER2-targeted therapies to access the brain, animal studies show that the BBB is likely compromised by brain lesions [17]. Moreover, in patients with HER2-positive breast cancer, accumulation of trastuzumab was 17.5-fold higher in brain metastases than in normal brain tissue [18]. As it is unclear what role access plays in treating brain metastases in patients with HER2-positive MBC, we investigated the extent of trastuzumab delivery, as well as efficacy of trastuzumab alone or in combination with a PI3K (phosphatidylinositol 3-kinase) inhibitor, and T-DM1 in experimental models of HER2-positive brain lesions.

## Methods

### Materials

Trastuzumab, T-DM1, muMAb 4D5 (the murine parent molecule of trastuzumab), control antibodies, and GNE-317 were from Genentech, Inc. ^89^Zr-labeled antibodies were synthesized as described [19]. Anti-STEAP1 was used as the isotype-matched control antibody for trastuzumab, and anti-CD22-DM1 served as the non-targeted control antibody–drug conjugate (ADC) for T-DM1. MMTV-human HER2 transgenic mice were established previously [20]. Tumors were obtained from the Fo2-1282 and Fo5 human HER2 transgenic lines for model development.

## Experimental Design and Procedures

### Fo2-1282 and Fo5 brain implant and orthotopic models

Immune-competent female FVB mice (age 6–8 weeks) were used in all experiments. Tumors from MMTV-human HER2 transgenic lines Fo2-1282 and Fo5 were propagated and maintained by serial orthotopic engraftment in the number 2/3 mammary fat pad in FVB mice. For inoculation into the brain, orthotopic tumors were harvested and dissociated to produce a single-cell suspension. The suspension containing cells from Fo2-1282 tumors was stereotactically injected into the right striatum of mice (250,000 cells in 5 µL BSA/PBS) under isoflurane anesthesia. Stereotaxic coordinates were AP +0.2–0.5 mm from bregma; M-L = 2 mm; D-V = 3.5-mm flat skull. The same procedure was followed to create experimental Fo5 brain grafts, except that 200,000 cells were inoculated. Sham surgery was performed using vehicle with no cells. Orthotopic tumors were established by surgically implanting 2 × 2 mm tumor fragments into the number 2/3 mammary fat pads. Hematoxylin and eosin (H&E) and immunohistochemical staining of HER2 were performed on brain grafts, as previously described [28]. Additional experimental details are provided in the online supplement. All studies were conducted in accordance with the Guide for the Care and Use of Laboratory Animals.

### Analysis of trastuzumab and muMAb 4D5 uptake into brain by ELISA

Trastuzumab (30 mg/kg) and muMAb 4D5 (10 or 30 mg/kg) were administered by intraperitoneal (IP) injection. Twenty-four hours after trastuzumab administration, blood samples were drawn from the left cardiac ventricle under anesthesia, vasculature was flushed with an intra-cardiac perfusion of saline solution maintained at 4 °C, and brain samples were harvested. Mice that did not undergo surgery served as additional controls. Trastuzumab and muMAb 4D5 were quantified in harvested samples using ELISA, the details of which are provided in the online supplement.

### Imaging

ImmunoPET was performed using ^89^Zr-labeled trastuzumab and ^89^Zr-labeled control anti-STEAP1 antibody, as described previously [19]. Uptake was quantified in brain tumor grafts, normal brain, liver, and blood (*n* = 2 for all groups). Predicted MuMAb 4D5 uptake in brain and orthotopic grafts was estimated by multiplying ^89^Zr-trastuzumab uptake with the injected dose of muMAb 4D5 (*n* = 10–12 per group). Antibody uptake was assessed between days 1–5 post-injection. Magnetic resonance imaging (MRI) was performed as previously described [21]. See online supplement for additional details.

### Statistical analysis of imaging studies

Plots were constructed with R software version 2.10.1 (R Foundation for Statistical Computing, Vienna, Austria). Statistical significance was determined using Student’s *t* test. *P* values less than 0.05 were considered significant; data are presented as mean ± standard deviation.

### Efficacy experiments in Fo2-1282 brain and mammary grafts and Fo5 brain grafts

To evaluate the efficacy of muMAb 4D5 in Fo2-1282 mammary fat pad versus brain tumor grafts, mice were administered 3, 10, 20, or 30 mg/kg antibody IP (*n* = 8 per group) after a 2× loading dose initially and once per week for 3 weeks at the indicated dose. Antibody diluent (8.6 g/L NaCl, 0.289 g/L sodium acetate, 0.086 mL/L polysorbate 20) was used as vehicle control. To investigate the efficacy of muMAb 4D5 combined with the brain-penetrant PI3K/mTOR inhibitor GNE-317 [22], muMAb 4D5 was given at a dose of 30 mg/kg weekly IP after a 60-mg/kg loading dose; GNE-317 was administered by oral gavage daily for 20 days at a dose of 30 mg/kg (*n* = 12 for each treatment group). For the Fo5 model, mice received a single intravenous (IV) dose (10 mg/kg) of either T-DM1 or non-targeted control ADC 9 days after inoculation of Fo5 tumor cells. Efficacy in mice bearing orthotopic mammary tumors was determined by caliper tumor volume measurement using the following formula: Tumor volume (mm^3^) = (Length × Width^2^) × 0.5. When tumors reached 200–400 mm^3^, mice were randomized into treatment groups. Data collected from each experimental group were expressed as mean ± standard error of the mean. Kaplan–Meier plots were generated for time-to-progression, defined as either time-to-tumor doubling from day 0 or survival if no tumor volume doubling occurred. Efficacy in mice bearing brain grafts was assessed by survival, defined as the length of time elapsing from the date of surgical cell injection to the date on which mice exhibited a ≥20% reduction in body weight and/or became moribund. The duration of survival was analyzed using Kaplan–Meier methodology. Statistical analysis was performed on all Kaplan–Meier curves using JMP software version 6.0 (SAS Institute).

## Results

### Characterization of the Fo2-1282 brain lesion model

**Fig. 1:**
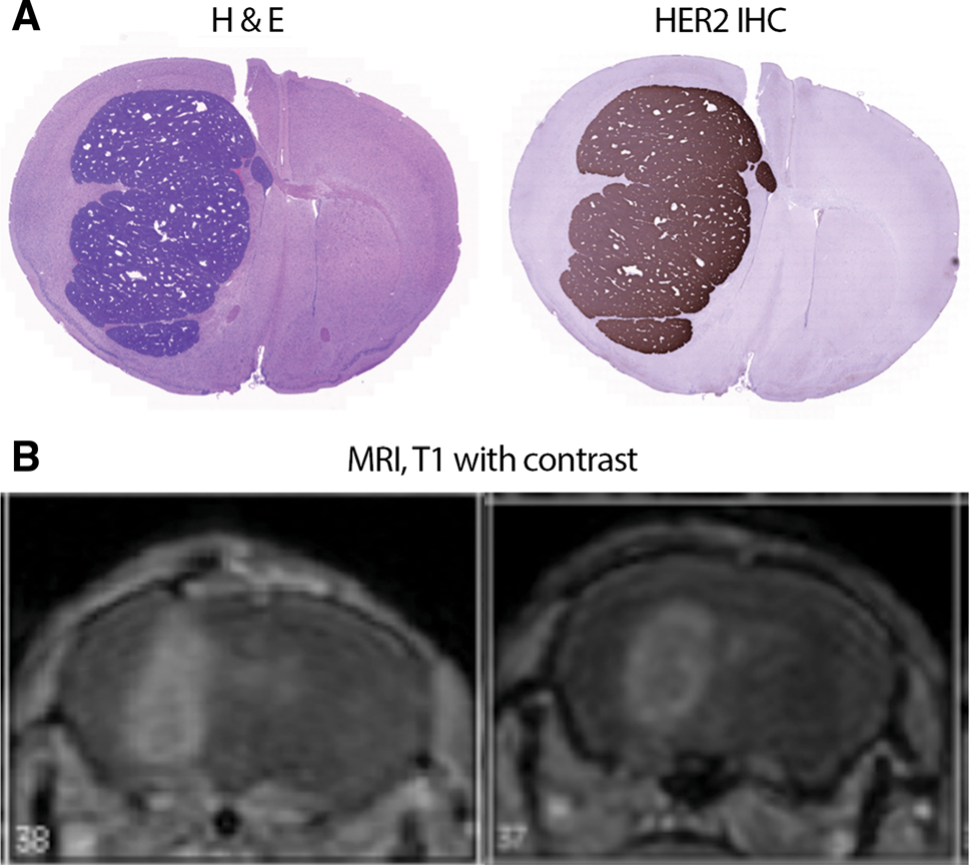
Characterization of Fo2-1282 brain metastasis model. **a** Hematoxylin and eosin (H&E) staining (*left*) and HER2 protein expression determined by immunohistochemistry (*right*). **b** T1-weighted MRI with contrast in separate tumor-bearing mice. Representative data are shown

Fo2-1282 brain grafts exhibited aggressive growth in immune-competent mice, as all animals succumbed to tumors on day 15–20 following surgical inoculation of tumor cells. Immunohistochemistry demonstrated uniform staining of HER2 throughout the tumors (Fig. 1a, right), and the distribution of HER2 expression matched the extent of the tumor, as revealed by H&E staining (Fig. 1a, left) and T1-weighted MRI (Fig. 1b).

**Fig. 2:**
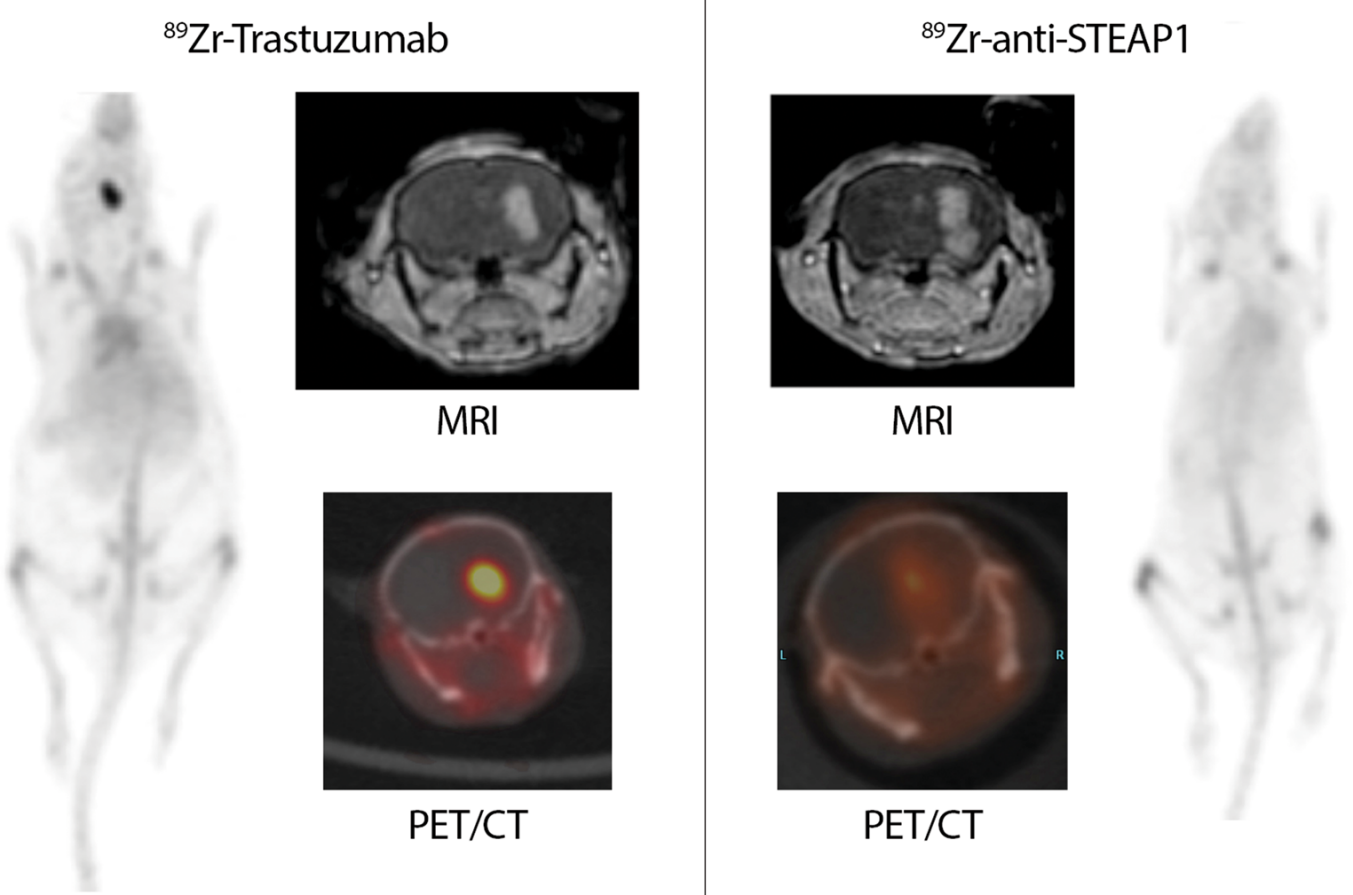
Trastuzumab uptake into Fo2-1282 brain grafts following a single systemic dose. PET imaging of ^89^Zr-trastuzumab (*left*) and ^89^Zr-anti-STEAP1 control antibody (*right*) uptake

**Fig. 3:**
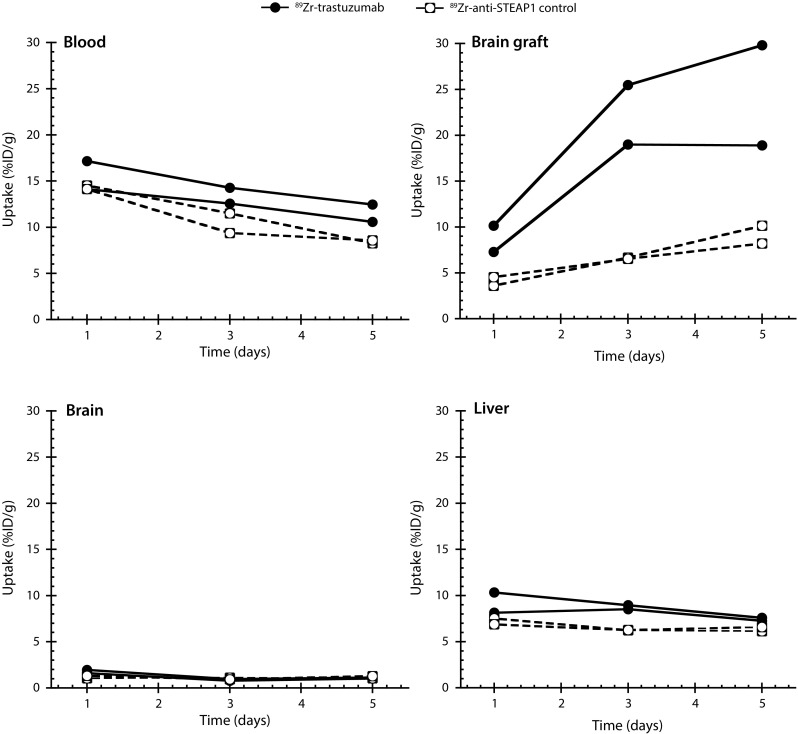
^89^Zr-trastuzumab uptake in brain lesions, normal brain, liver, and blood following a single systemic dose. Concentrations of ^89^Zr-trastuzumab were measured at 1, 3, and 5 days (*n* = 2 for each group; *each line* represents one animal)

To investigate the degree and specificity of trastuzumab uptake into Fo2-1282 brain grafts, tumor-bearing mice were administered a single systemic dose of either ^89^Zr-trastuzumab or control ^89^Zr-anti-STEAP1, then subjected to PET imaging. PET scans revealed robust uptake of ^89^Zr-trastuzumab into brain grafts (Fig. 2, left). Specificity of ^89^Zr-trastuzumab tumor uptake was demonstrated by the presence of only weak ^89^Zr-anti-STEAP1 signal in separate Fo2-1282 brain grafts (Fig. 2, right). The ^89^Zr-labeled antibodies were administered at day 14 post-inoculation, and PET images were acquired at 1, 3, and 5 days post-tracer injection. At all three time points, the concentration of ^89^Zr-trastuzumab within the brain graft was greater than that of control (Fig. 3). Uptake of ^89^Zr-trastuzumab in the brain graft was 24.4% ID/g (percent injected dose per gram tissue) at day 5 post-injection, but uptake of the control antibody reached only 9.2% ID/g. The apparent uptake in the contralateral side of the brain was 1.1% ID/g for ^89^Zr-trastuzumab and 1.2% ID/g for ^89^Zr-control antibody (Fig. 3). The uptake of both ^89^Zr-antibodies was also comparable in blood and liver (Fig. 3).

Gadolinium-contrast MRI revealed gadolinium leakage at the site of the brain graft (Fig. 2), suggesting that growth of Fo2-1282 brain lesions had locally compromised the BBB. Compared with the contralateral side, uptake of control antibody in the lesion was elevated, a likely consequence of the compromised BBB (Fig. 3). Concentrations of the two labeled antibodies were similar in blood and non-malignant tissues, including normal brain and liver (Fig. 3), further demonstrating specific uptake of ^89^Zr-trastuzumab into Fo2-1282 brain grafts. These results show that the contrast-enhanced lesions are accessible to targeted antibody penetration, as measured with radiolabeled trastuzumab.

**Fig. 4:**
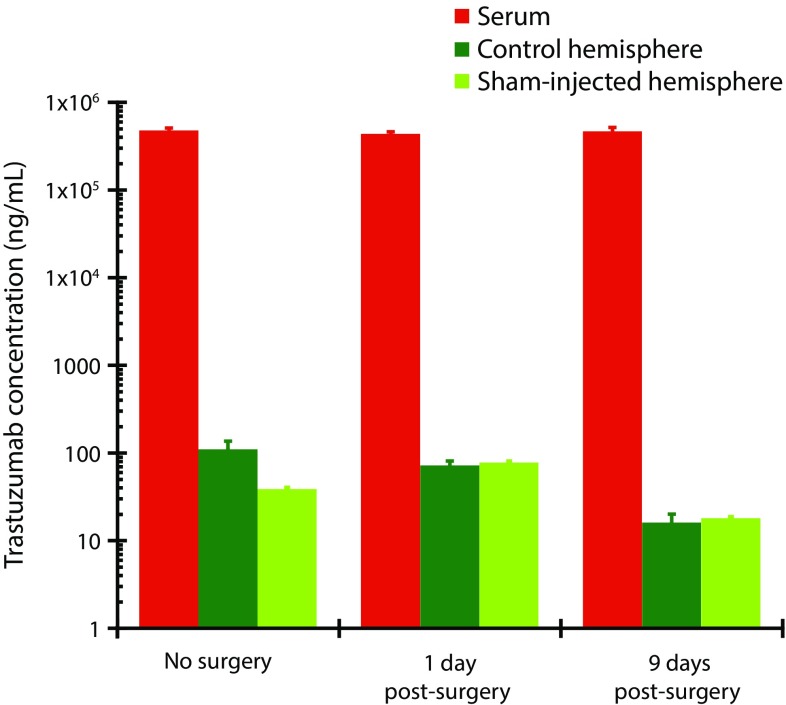
No impact of stereotactic surgery on trastuzumab concentrations in brain following systemic delivery. Mice received sham surgery in one hemisphere and no surgery in the other. Trastuzumab was measured by ELISA in each brain hemisphere and in serum at 1 (*n* = 4) and 9 (*n* = 4) days after these procedures

To determine whether stereotactic surgery was itself responsible for the prolonged disruption of the BBB (thereby permitting increased uptake of trastuzumab into brain grafts), trastuzumab was systemically administered to animals that had received sham injection in one brain hemisphere and no injection (control) in the other hemisphere. Concentrations of trastuzumab were 1000-fold lower in the sham-injected brain hemisphere than in serum (Fig. 4). The concentration of trastuzumab in the sham-injected hemisphere was similar to that in the non-injected control hemisphere (Fig. 4). These results did not markedly differ between day 1 (pre-surgical wound healing) and day 9 (post-healing) post-surgery. Together, these findings suggest that the physical trauma of stereotactic brain surgery did not lead to increased uptake of trastuzumab in the brain.

**Fig. 5:**
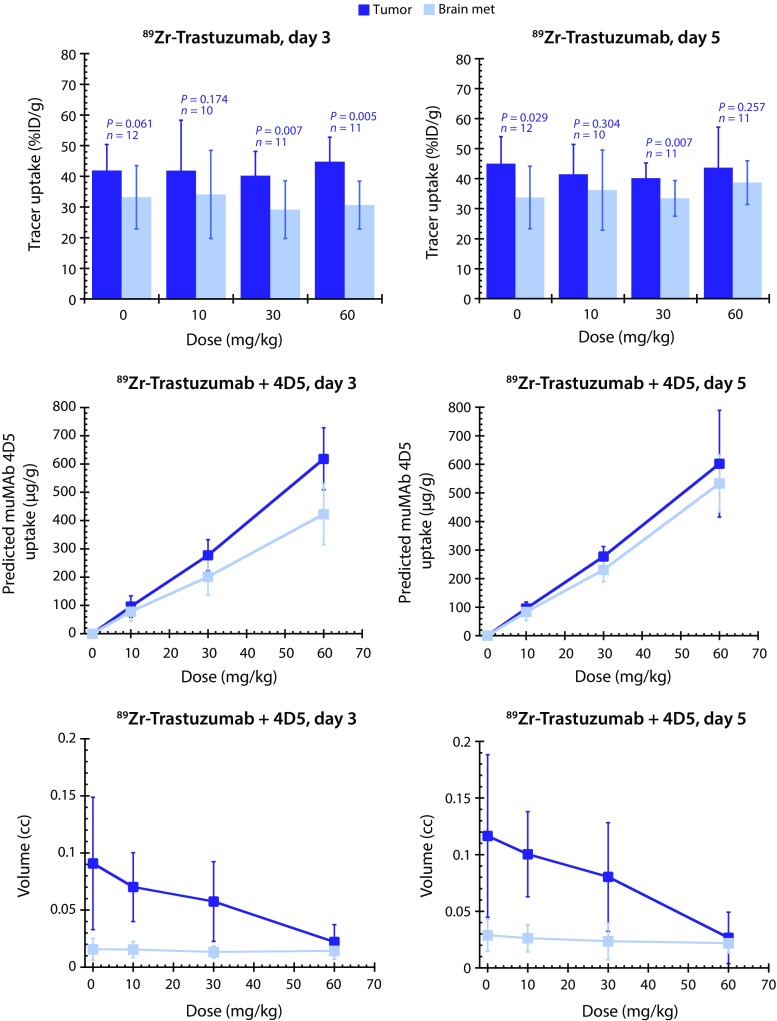
^89^Zr-trastuzumab uptake in Fo2-1282 brain and mammary tumors following a single systemic dose. Uptake of ^89^Zr-trastuzumab was assessed as  %ID/g (*top panels*); predicted muMAb4D5 quantification (μg/g) calculated from ^89^Zr-trastuzumab uptake × injected dose (*middle panels*), and volume (*lower panels*). *N* = 10–12 animals per group

The uptake of ^89^Zr-trastuzumab was subsequently compared between Fo2-1282 brain lesions and Fo2-1282 mammary fat pad (orthotopic) tumors. Nearly equivalent ^89^Zr-trastuzumab uptake was observed in brain grafts and mammary fat pad tumors at both day 3 and day 5 post-surgery (Fig. 5, top panels). Tracer was used to estimate muMAb4D5 uptake in brain and mammary fat pad lesions at a given dose (Fig. 5, middle panels). Orthotopic tumors showed strong dose-dependent decreases in tumor volume, as measured by MRI, at 3 and 5 days post-treatment (Fig. 5, bottom panels). Volume changes in the brain lesions were modest, due in part to limitations in determining volume of small tumors by imaging and to assessment at such early time points. However, there was a trend for tumor growth delay in the high-dose muMAb 4D5 group at day 5 (Supplementary Table 1).

### Efficacy of muMAb 4D5 in orthotopic mammary tumors and brain lesions

**Fig. 6:**
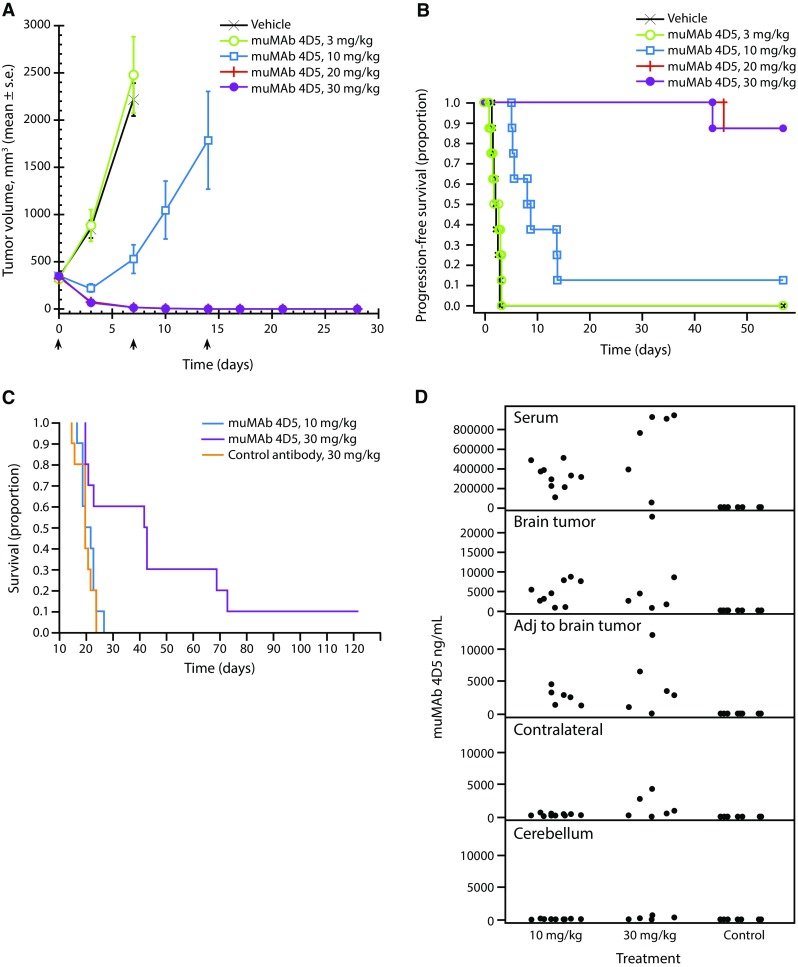
Efficacy of muMAb 4D5 in Fo2-1282 mammary tumors and brain lesions. Antibody treatments were weekly × 3, following a 2× loading dose, at doses of 3, 10, 20, or 30 mg/kg. **a** Orthotopic mammary tumor volume as determined by caliper measurement; **b** survival in mice bearing orthotopic mammary tumors; or **c**, brain lesions, as assessed using Kaplan–Meier analysis. *Arrows* denote day of antibody administration. **d** muMAb 4D5 concentrations were assayed in serum, brain graft, normal brain adjacent to graft, un-injected contralateral hemisphere, and normal cerebellum at end of study

muMAb 4D5, administered weekly for 3 weeks, showed dose-dependent anti-tumor activity in Fo2-1282 orthotopic mammary tumors (Fig. 6A), with the 20 and 30 mg/kg doses causing complete tumor regression in all animals, and cures in most mice as evidenced by survival beyond 50 days (Fig. 6b). Three weekly doses of muMAb 4D5 at 10 mg/kg caused tumor growth inhibition and extended survival in mice with mammary tumors. The increased survival in all treatment groups differed significantly from the vehicle control group (log-rank *p* < 0.0001 for vehicle versus 10, 20, and 30 mg/kg groups).

The efficacy of muMAb 4D5 was, however, markedly lower against Fo2-1282 brain lesions. Administration of muMAb 4D5 doses of 10 mg/kg weekly did not extend survival relative to vehicle-treated control (Fig. 6c), with all mice succumbing by day 26. Administration of 30 mg/kg weekly for 3 weeks was efficacious, as demonstrated by increased survival up to 70 days (Fig. 6c). However, in contrast to the complete regressions demonstrated in the mammary tumor model, cures were not observed in animals with experimental brain lesions.

Tissue concentrations of muMAb 4D5 were then quantified to determine whether lack of uptake into Fo2-1282 brain grafts was responsible for the limited efficacy. For both the 10 mg/kg and 30 mg/kg doses of muMAb 4D5, antibody concentrations within the brain grafts were 1000–10,000 ng/mL—concentrations known to have anti-proliferative activity in vitro [23]—suggesting that decreased efficacy was not due to limited access of the antibody to brain lesions (Fig. 6d).

### Activity of combination therapy in experimental brain metastasis model

**Fig. 7:**
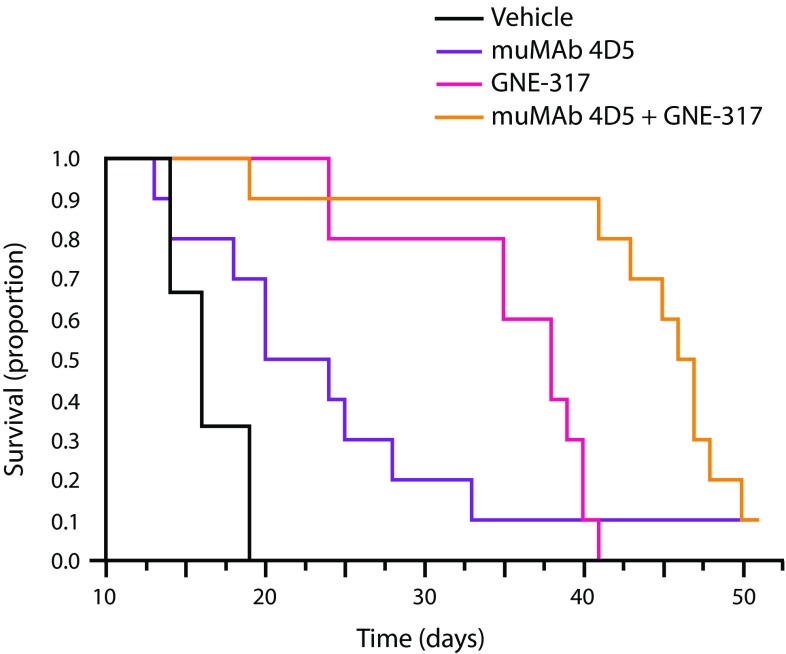
Enhanced survival effect of muMAb 4D5 combined with GNE-317 versus single-agent treatment in mice bearing Fo2-1282 brain lesions. Mice were administered muMAb 4D5 IV weekly (30 mg/kg following a 2× loading dose) and/or 30 mg/kg GNE-317 daily by oral gavage. Treatment was initiated on day 10 and terminated on day 30. *Arrows* denote antibody treatment; *solid line denotes* GNE-317 treatment

PI3K signaling is constitutively active in HER2-amplified cancer cells. Preclinical studies have demonstrated enhanced anti-tumor activity by combining PI3K inhibitors with trastuzumab [24]. A brain-penetrant PI3K/mTOR inhibitor, GNE-317, was shown to have anti-tumor activity in orthotopic models of glioblastoma [22]. We therefore investigated combining GNE-317 with muMAb 4D5 in the Fo2-1282 brain lesion model. Mice with Fo2-1282 brain grafts were administered vehicle only or one of three treatments: muMAb 4D5 alone (30 mg/kg weekly × 3 after 2× loading dose), GNE-317 alone (30 mg/kg daily), or both agents. Single-agent muMAb 4D5 and single-agent GNE-317 each extended survival relative to vehicle control. However, survival with the combination of muMAb 4D5 and GNE-317 was greater than either single-agent treatment (Fig. 7).

### Effect of T-DM1 on survival in mice bearing experimental brain lesions

**Fig. 8:**
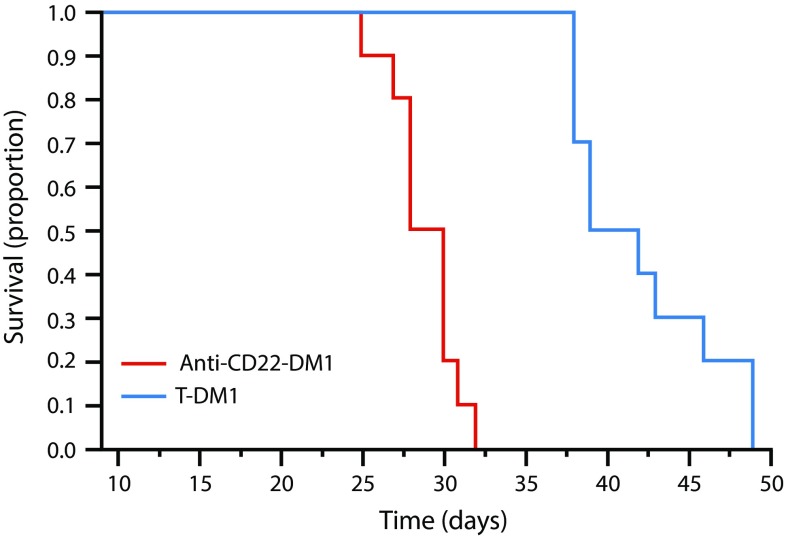
Enhanced survival in mice bearing Fo5 brain lesions after treatment with a single dose of 10 mg/kg T-DM1 compared with 10 mg/kg of non-targeted control ADC

After establishing anti-tumor activity of muMAb 4D5/trastuzumab in the Fo2-1282 brain graft model, it was of interest to determine efficacy of T-DM1, an additional approved HER2-targeted therapeutic agent. For this purpose, a trastuzumab-insensitive HER2 transgenic tumor line, Fo5, was utilized [25]. A single IV dose of 10 mg/kg T-DM1 or control ADC (anti-CD22-DM1) was administered to mice bearing Fo5 intracranial grafts. As shown in Fig. 8, T-DM1 extended both median (50%) and overall survival of these brain lesion-bearing mice by approximately 2 weeks relative to control-treated animals.

## Discussion

In retrospective clinical studies, trastuzumab was demonstrated to prolong OS in patients with brain metastases from HER2-positive MBC [4, 8, 12]. It remains unclear if increased OS is due to effective control of systemic, extra-cranial disease or a more direct effect on brain metastases [4, 12]. A key outstanding question has been to what degree access to the brain impacts the efficacy of trastuzumab in these lesions.

Although the Fo5 and Fo2-1282 models involve direct intracranial injection of tumor cells, rather than seeding of brain lesions from systemic circulation, these models were selected for evaluation of HER2-targeted agents in established brain metastases. Steeg et al. [3] demonstrated the involvement of HER2 signaling in outgrowth of breast cancer-derived experimental brain metastases, but not in initiation of these lesions [10]; outgrowth therefore appears to be the key process to capture in a model used to evaluate the effects of HER2 inhibition. This outgrowth process is well represented in the Fo5 and Fo2-1282 models.

The Fo5 and Fo2-1282 models used in our experiments also differ from those utilized by Steeg et al. in a significant way: while the model tumors based on 231-BR cells are grown in athymic nude mice [26, 27], our models use immune-competent mice and therefore lend themselves particularly well to evaluating the efficacy of trastuzumab, an antibody whose mechanism of action includes the recruitment of immune effector cells and activation of antibody-dependent cell-mediated cytotoxicity or ADCC [28]. Overall, the efficacy results reported here are similar to those obtained by Steeg et al. [3] with another HER2-targeted agent, lapatinib, in an animal model of breast cancer-derived brain metastasis. As with trastuzumab, lapatinib inhibited metastatic outgrowth of brain lesions at high doses, but inhibition was incomplete [26].

In the Fo2-1282 mouse model of HER2-positive breast cancer-derived brain metastases, 3-fold higher systemic doses of muMAb4D5/trastuzumab were required to achieve efficacy in brain tumor grafts compared with those in the mammary fat pad. The reduced efficacy of muMAb4D5 in treating brain grafts did not appear to result from lack of access, as PET imaging showed ^89^Zr-trastuzumab to localize equivalently in brain and mammary grafts. Furthermore, ^89^Zr-trastuzumab localization in the HER2-positive tumor graft was significantly greater compared to normal brain tissue and muMAb 4D5 was demonstrated to accumulate in Fo2-1282 brain grafts at known therapeutic concentrations.

There are several hypotheses put forward as to the reduced efficacy of trastuzumab in Fo2-1282 brain lesions compared with mammary tumors: the inability of immune effector cells to access the brain lesion, thereby impairing ADCC; the presence of ErbB ligands in the brain microenvironment circumventing HER2 inhibition by trastuzumab; or activation of compensatory signaling pathways. Although we did not directly investigate the access of effector cells to brain grafts, previous reports show the activity of immune cell-dependent therapies in preclinical models of glioma [29], as well as in patients with melanoma brain metastases [30]. These observations are consistent with our hypothesis that lack of access to effector cells does not explain reduced trastuzumab efficacy in brain grafts.

Our results further suggest that the reduced efficacy of muMAb 4D5 in Fo2-1282 brain grafts compared with mammary tumors may arise from incomplete pathway suppression or hyper-activation of downstream signal transduction pathways, as combined treatment with muMAb 4D5 and the brain-penetrant PI3K/mTOR inhibitor GNE-317 was more effective than either drug alone. One possible explanation for the diminished muMAb 4D5 response is the presence of brain-specific ligands that mediate resistance to HER2 inhibition. Multiple redundant HER family ligands mediate insensitivity to trastuzumab or other HER2-targeted agents [31], and it is likely that signaling driven by these ligands converges on important downstream cell-survival pathways such as PI3K. Recent reports suggest that resistance to anti-cancer tyrosine kinase inhibitors is frequently triggered by the presence of additional receptor tyrosine kinase ligands [31], and in vitro experiments suggest that ligand-driven activation of alternative receptors in the HER family may present a recurrent mechanism of resistance in breast cancer cells [32].

An alternate approach to circumventing insensitivity to anti-HER2 therapies is to target a potent cytotoxic agent to HER2-positive tumors by utilizing a HER2-directed ADC. T-DM1 was demonstrated to have superior anti-tumor activity compared with trastuzumab in HER2-positive preclinical models [25]. Improved survival after T-DM1 treatment was demonstrated in mice with experimental lesions from the Fo5 model, a model that does not respond to trastuzumab [25]. In the phase III EMILIA trial of patients with HER2-positive MBC previously treated with trastuzumab and a taxane, T-DM1 showed improved PFS and OS compared with lapatinib plus capecitabine [33]. Importantly, in a subset of EMILIA study participants with asymptomatic CNS metastases at baseline, T-DM1 was associated with significantly improved survival compared with lapatinib and capecitabine [34].

In conclusion, our data provide a rationale for the clinical evaluation of higher-dose trastuzumab, T-DM1, or combination therapy with two or more targeted agents for the treatment of brain metastases in patients with HER2-positive MBC. To this end, a trial designed to assess the efficacy of high-dose trastuzumab, combined with pertuzumab, in patients with HER2-positive MBC and CNS progression post-radiotherapy (NCT02536339) is currently enrolling patients.

## Electronic supplementary material

Below is the link to the electronic supplementary material.

Supplementary material 1 (PDF 93 kb)

Supplementary material 2 (DOCX 13 kb)

## Abbreviations

ADC: Antibody-drug conjugate
ADCC: Antibody-dependent cell-mediated cytotoxicity
BBB: Blood–brain barrier
BSA: Bovine serum albumin
CNS: Central nervous system
ELISA: Enzyme-linked immunosorbent assay
H&E: Hematoxylin and eosin staining
HER2: Human epidermal growth factor receptor 2
IP: Intraperitoneal
IV: Intravenous
MBC: Metastatic breast cancer
MRI: Magnetic resonance imaging
OS: Overall survival
PBS: Phosphate-buffered saline
PET: Positron emission tomography
PI3K: Phosphatidylinositol 3-kinase
T-DM1: Trastuzumab emtansine
