# Supplementary material for: Trastuzumab uptake and its relation to efficacy in an animal model of HER2-positive breast cancer brain metastasis
Source: Breast Cancer Res Treat. 2017 May 10;164(3):581–91. doi: 10.1007/s10549-017-4279-4 (PMC5495871; doi:10.1007/s10549-017-4279-4)
Supplement: Supplementary file 1 — Supplementary material 1 (PDF 93 kb) [file 10549_2017_4279_MOESM1_ESM.pdf]

Supplementary Material

Trastuzumab uptake and its relation to efficacy in an animal model of HER2-positive breast cancer brain metastasis

Breast Cancer Research and Treatment

Gail D. Lewis Phillips[1], Merry C. Nishimura, Jennifer Arca Lacap, Samir Kharbanda, Elaine Mai, Janet Tien, Kimberly Malesky, Simon P. Williams, Jan Marik, Heidi S. Phillips

[1]Corresponding author:

Genentech, Inc.

1 DNA Way

South San Francisco, CA 94080

Email: gdl@gene.com

Experimental Design and Procedures

Fo2-1282 and Fo5 brain implant and orthotopic models

Tumors from MMTV-human HER2 transgenic lines Fo2-1282 and Fo5 were propagated and maintained by serial orthotopic engraftment into the number 2/3 mammary fat pad in FVB mice. For inoculation into brain, tumors were harvested and disassociated to produce a single-cell suspension. The tumor was cut into small fragments, immersed in an enzymatic solution (2.5 mg/mL each of Type II and Type IV collagenase, 0.5 mg/mL DNAse and 3 mM $CaCl_2$ in 1% BSA/PBS) and incubated for 15 min in a 37°C shaker. The tumor mixture was then passed through a 70-µm strainer, and after several washes

in BSA/PBS, cell number was determined. The suspension containing cells from Fo2-1282 tumors was stereotactically injected into the right striatum of mice (250,000 cells, in 5 μL BSA/PBS) under isoflurane anesthesia. Cells were infused at a rate of 1 μL/min using a 10-μL Hamilton syringe fitted with a 28-g stainless steel cannula. After infusion, the cannula was left in place for 3–5 min, then retracted at 2 mm/min. Stereotaxic coordinates were AP +0.2–0.5 mm from bregma; M-L = 2 mm; D-V = 3.5 mm flat skull. The same procedure was followed to create experimental Fo5 brain grafts, except that 200,000 cells were inoculated.

Analysis of trastuzumab and muMAb 4D5 uptake into brain by ELISA

To measure total trastuzumab or muMAb 4D5, HER2 extracellular domain was coated onto 384-well maxisorp NUNC plates. Captured total trastuzumab was detected with goat anti-human Fc conjugated to horseradish peroxidase (HRP); captured muMAb 4D5 was detected with goat anti-murine Fc HRP conjugate (both from Jackson ImmunoResearch Laboratories). The limit of quantitation (LOQ) for trastuzumab in brain tissue homogenate was 41 pg/mL and for mouse serum was 205 pg/mL. The LOQ for muMAb 4D5 in brain tissue homogenate was 2 ng/mL and for mouse serum was 20.5 ng/mL.

^{89}Zr-ImmunoPET imaging and image analysis

Fo2-1282 cells or tumor fragments were inoculated into brain or mammary fat pad, respectively. Mice bearing paired grafts in brain and mammary fat pad were administered muMAb 4D5 (10, 30, or 60 mg/kg) via intraperitoneal injection (*n* = 6 per group). The following day, mice were administered intravenous ^{89}Zr-trastuzumab (3

mg/kg). PET scans were performed to measure uptake of ^{89}Zr-trastuzumab into brain and breast tumor grafts on day 3 and day 5 following administration.

MRI imaging

Animals were anesthetized and maintained on 1.5–2% isoflurane in medical air. Body temperature was monitored and maintained at 37°C ± 0.5°C using warm air. After a pilot scan to confirm positioning, a fast spin echo sequence (field of view 20 mm^2; slice thickness 0.8 mm; matrix 128^2 zero-filled to 256^2; TR 4 s; TE_{eff} 36 ms; echo train length 8; echo spacing 9 ms; NEX 8) was used to acquire coronal data sets at 4.7 T (Direct Drive console, Varian Inc., Palo Alto, CA) using a 3-cm inner diameter radio frequency transmit/receive volume coil (Millipede, Varian Inc., Palo Alto, CA). The image analysis to obtain volumetric measurements was performed using Analyze 8.1 (Biomedical Imaging Resources, Rochester, MN).

Animals were administered gadolinium diethylenetriamine penta-acetic acid (Gd-DTPA) contrast agent (Omniscan, GE Healthcare, Oslo, Norway) via a tail vein catheter at a dose of 0.1 mmol Gd/kg. A three–dimensional gradient echo sequence was used to qualitatively assess tumor enhancement (field-of-view 20 mm^3; slab thickness 15 mm; matrix 64 × 64 × 32 zero-filled to 128 × 128 × 64; flip angle 10°; repetition time 6 ms; echo time 2 ms; number of excitations 4; six data sets acquired post-contrast injection; total scan time 10 min).
