# Supplementary material for: Trastuzumab uptake and its relation to efficacy in an animal model of HER2-positive breast cancer brain metastasis
Source: Breast Cancer Res Treat. 2017 May 10;164(3):581–91. doi: 10.1007/s10549-017-4279-4 (PMC5495871; doi:10.1007/s10549-017-4279-4)
Supplement: Supplementary file 2 — Supplementary material 2 (DOCX 13 kb) [file 10549_2017_4279_MOESM2_ESM.docx]

**Supplementary Table 1. Brain lesion volumes (cc) 3 days and 5 days after administration of ^89^Zr-trastuzumab (*n* = 10-12 per group)**

| **Dose (mg/kg)** | **Volume (cc) day 3** | **Volume (cc) day 5** | **P-value** |
| --- | --- | --- | --- |
| vehicle control | 0.01564 | 0.02886 | 0.014 |
| 10 | 0.01551 | 0.02621 | 0.028 |
| 30 | 0.01316 | 0.02348 | 0.069 |
| 60 | 0.01425 | 0.02191 | 0.040 |
